# Fighting antimicrobial resistance in Brazil: strengthening diagnostic stewardship, antimicrobial stewardship, and policies for a healthier future

**DOI:** 10.3389/fpubh.2025.1726000

**Published:** 2026-01-16

**Authors:** Marcelo Carneiro, Marcelo Pillonetto

**Affiliations:** 1 Department of Life Sciences, School of Medicine, Postgraduate Program in Health Promotion, University of Santa Cruz do Sul, Santa Cruz do Sul, Brazil; 2 Brazilian Association of Professionals in Infection Control and Hospital Epidemiology, Brasília, Brazil; 3 School of Medicine and Health Sciences, Pontifical Catholic University of Paraná, Curitiba, Brazil; 4 Bacteriology Division, Central Laboratory of the State of Paraná, Curitiba, Brazil

**Keywords:** antimicrobial resistance, antimicrobial stewardship, Brazil, diagnostic stewardship, healthcare economics, Latin America, One Health, public health policy

## Abstract

Antimicrobial resistance (AMR) constitutes a growing public health crisis in Latin America, with Brazil facing particularly severe challenges. The drivers are multifaceted, prominently including suboptimal antimicrobial prescribing practices. Limited access to quality diagnostics and weak implementation of DS and AMS programmes further impede timely antimicrobial de-escalation and worsen the problem. National surveys confirm this gap; one 2019 survey of 954 hospitals with ICUs found that only 47.5% had implemented an ASP. This review critically examines the current landscape of AMR in Brazil, analyzing policy gaps and implementation challenges across primary, secondary, and tertiary healthcare sectors within the public, supplementary, and private healthcare systems. Key findings underscore the imperative to enhance diagnostic stewardship for optimizing antimicrobial selection, bolster surveillance systems, which now reveal a concerning rise in *bla*_NDM_ carbapenemases, and reinforce AMS programs across all healthcare settings. Strategic recommendations emphasize crucial investments in laboratory infrastructure and rapid diagnostic technologies, the adoption of value-based healthcare policies to overcome reimbursement barriers, and to incentivize quality outcomes over quantity, and the cultivation of robust regional and international collaborations to AMR effectively. Finally, it recommends the implementation of a National Policy Statement, as well as a National Program on Antimicrobial Resistance Prevention and Control.

## Graphical abstract

**Figure:**
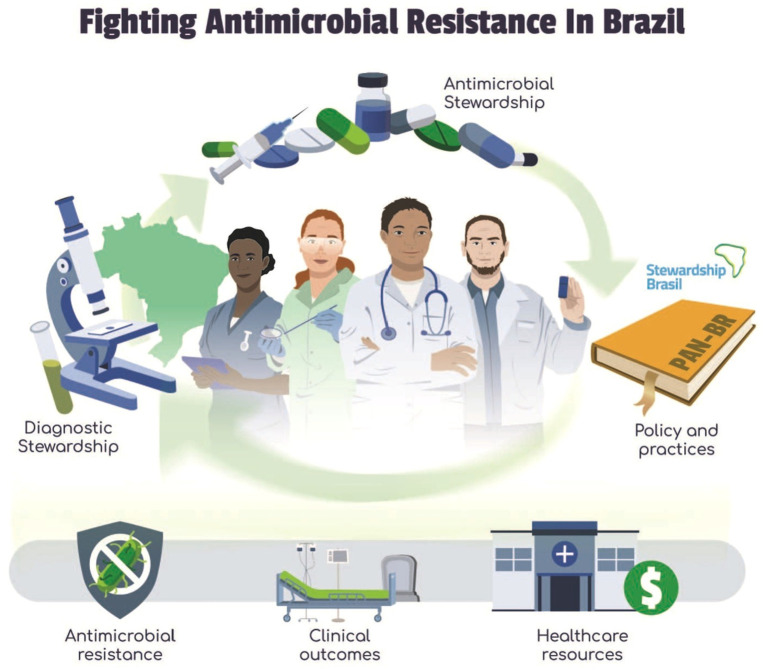
Illustrations are original works created by MIND (https://www.mind.net.br/).

## 1 Introduction

Antimicrobial resistance (AMR) represents one of the most significant global health threats of the 21st century, undermining decades of medical progress and posing substantial risks to public health and economic stability, affecting human, animal, and environmental health as well as food security (1). It is believed that without targeted investments in prevention and control, global life expectancy could decrease by up to 1.8 years by 2035, with a decline of up to 2.3 years in the Americas.

In Brazil, there are 33,200 direct deaths and 137,900 deaths associated with AMR each year (2). Thus, AMR impacts the Sustainable Development Goals (SDGs) established by the United Nations General Assembly, especially SDG 3 (Good Health and WellBeing) (3), a core component of Brazil’s national commitment to the 2030 Agenda (4). The efficacy of antibiotics, cornerstone treatments for countless infections, is diminishing at an alarming rate due to the emergence and spread of resistant microorganisms.

Latin America (LATAM) is a region experiencing a particularly high burden of AMR, with Brazil, its largest nation, at the forefront of this challenge. The escalating prevalence of multidrug-resistant organisms (MDROs), particularly carbapenem-resistant organisms (CROs), poses significant clinical and public health challenges (2, 4–8).

The drivers of resistance in LATAM and Brazil are complex and interconnected. High rates of carbapenemase enzymes, including New Delhi Metallo-*β*-lactamase (NDM), *Klebsiella pneumoniae* Carbapenemase (KPC), and various oxacillinases (e.g., OXA-23), are widespread among critical Gram-negative pathogens such as Enterobacterales, *Pseudomonas aeruginosa,* and *Acinetobacter baumannii* (8–16).

Data from Brazil reveal a stark increase in the detection of *bla*_NDM_ genes in Enterobacterales, rising from 4.2% in 2015 to an alarming 23.8% in 2022, a trend significantly exacerbated by the healthcare pressures and disruptions associated with the COVID-19 pandemic (8).

Infections caused by resistant pathogens make treatments more expensive, prolonged, and less accessible, especially in low- and middle-income countries (LMIC) (4). As a result, this scenario not only limits therapeutic options but also contributes to increased morbidity, mortality, and longer hospital stays.

The economic consequences of AMR are profound. Increased treatment costs, the need for more expensive second-line therapies, extended hospitalizations, and productivity losses impose a heavy burden on healthcare systems and national economies. In a scenario of high-AMR impact, by 2030, the global GDP could be reduced by 3.8% resulting in a US$3.4 trillion loss due per year, and 28 million people may be pushed into extreme poverty by 2050. The situation is even more dramatic for LIC and LMIC, like most countries in Latin America and Brazil, where the GDP loss could reach −4.4 to −5.1% (17).

A 2024 report indicates that infections caused by multidrug-resistant organisms (MDROs) could result in over 39 million human fatalities between 2025 and 2050. Furthermore, the economic repercussions of drug resistance in livestock are projected to incur losses of up to $950 billion to global GDP, while the transmission of these pathogens from livestock to humans could lead to costs as high as $5.2 trillion. The most impoverished populations globally, particularly those residing in LMIC, are disproportionately susceptible to these impacts (18).

**Figure 1:**
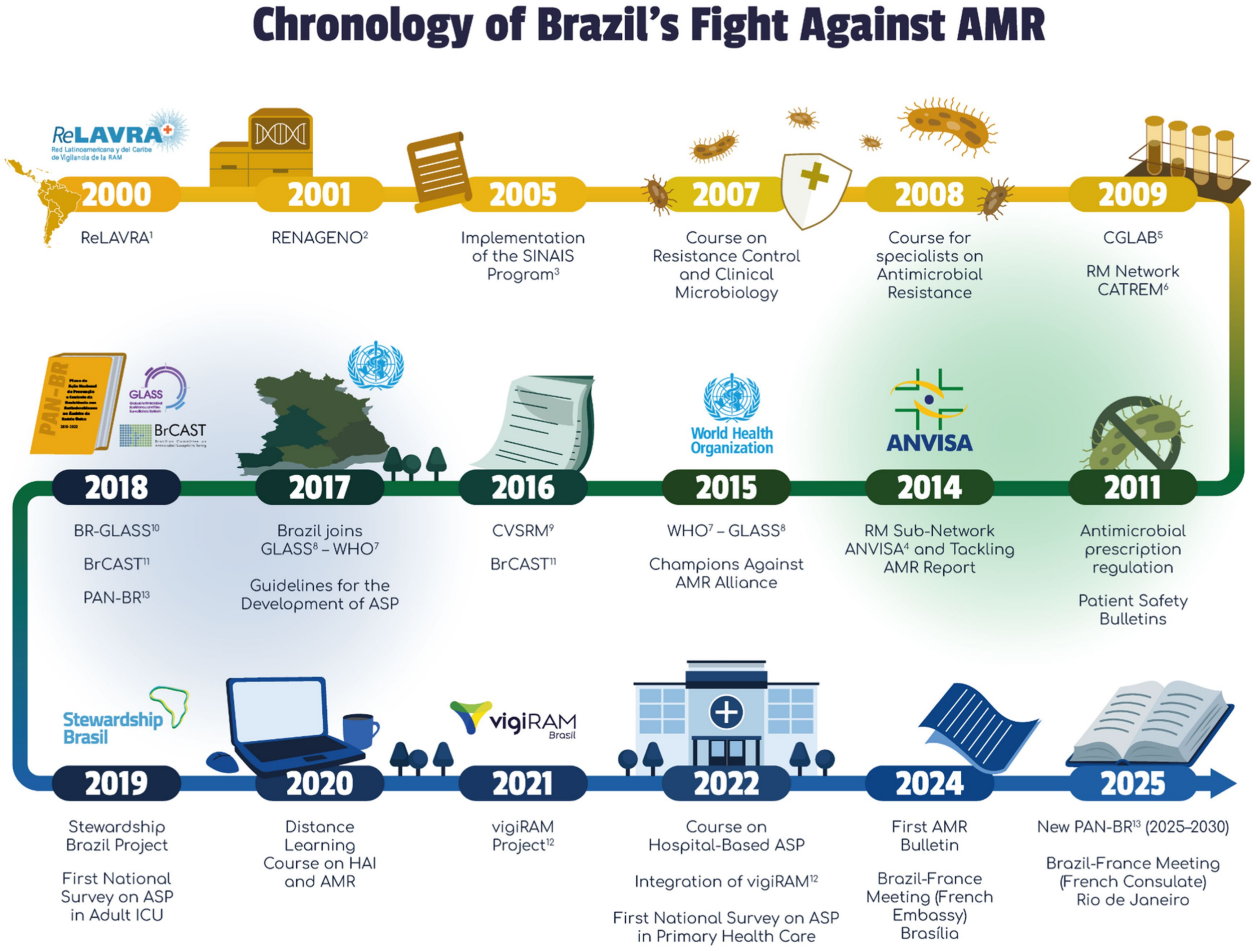
Brazil’s journey in combating AMR: a timeline of critical developments. RM, microbial resistance; AMR, antimicrobial resistance; ASP, Antimicrobial Stewardship Program; HAI, Healthcare-Associated Infection; ICU, intensive care unit; ^1^RELAVRA—Latin American Antimicrobial Resistance Surveillance Network; ^2^RENAGENO—National Genotyping Network; ^3^SINAIS—Program - National Health Care-Associated Infection Control Information System; ^4^ANVISA—National Health Surveillance Agency; ^5^CGLAB/MS—General Coordination of Public Health Laboratories at Minister of Health; ^6^CATREM—Technical Chamber for Antimicrobial Resistance in Health Care Services; ^7^WHO—World Health Organization; ^8^GLASS—Global Antimicrobial Resistance and Use Surveillance System; ^9^CVSRM—Commission for Health Surveillance on Antimicrobial Resistance; ^10^BR-GLASS—Brazilian Chapter, Global Antimicrobial Resistance and Use Surveillance System; ^11^BrCAST—Brazilian Committee on Antimicrobial Susceptibility Testing; ^12^vigiRAM Project—Strengthening a Brazilian Antimicrobial Resistance Surveillance System; ^13^PANBR—National Action Plan (NAP) for the Prevention and Control of Antimicrobial Resistance in Brazil. Illustrations are original works created by MIND (https://www.mind.net.br/).

Pathogens identified by the World Health Organization (WHO) as critical priorities for research and development of new antibiotics, such as carbapenem-resistant Enterobacterales, *A. baumannii*, and *P. aeruginosa*, are highly prevalent throughout Latin America (LATAM) (6, 7, 13, 16, 19). Addressing this requires urgent action, yet the region often grapples with limited resources, fragmented healthcare systems, and inconsistencies in AMR surveillance and control efforts. The emergence and escalating burden of AMR in Brazil have necessitated a progressive and structured public health response, the chronology of which underscores the nation’s commitment to combating this threat, whose details are shown in Figure 1.

The foundational efforts began in the early 2000s with the joining of the Latin American Antimicrobial Resistance Surveillance Network (ReLAVRA) in 2000 (20). A significant policy milestone occurred in 2011 when the Brazilian Health Regulatory Agency (ANVISA) implemented the regulation of antimicrobial prescription, transitioning these drugs from over-the-counter sales, an essential step for stewardship.

AMR surveillance framework was strengthened in 2017 when Brazil officially joined the WHO Global Antimicrobial Resistance and Use Surveillance System (GLASS), aligning its national surveillance with global standards (1, 21, 22). Subsequent years saw the introduction of the National Action Plan (PAN-BR) in 2018 (23) and the launch of the Stewardship Brasil Project in 2019 (24), marking a concerted effort to integrate policy and practice, notably in adult intensive care units (ICUs). The most recent developments highlight an evolution toward continuous refinement and global engagement, including the launch of the vigiRAM Project in 2021 to enhance laboratory capacity and monitoring and the planning of the updated New PAN-BR for 2025–2030, which will guide future multisectoral strategies (25). This sequence of events, depicted in Figure 1, demonstrates a continuous effort to strengthen surveillance, diagnostic, and stewardship programs in Brazil.

Recognizing the urgency, Brazil has implemented The National Action Plan for the Prevention and Control of Antimicrobial Resistance (PAN-BR) (26), which is aligned with the WHO Global Action Plan and the One Health approach, thereby integrating the human, animal, and environmental health sectors (21). However, significant gaps remain in translating policy into consistent practice across the diverse Brazilian healthcare landscape, encompassing the public Unified Health System (SUS), the supplementary health sector, and private providers (26–28).

This policy and practice review aims to provide a comprehensive analysis of the AMR situation in Brazil, integrating evidence from the scientific literature, expert insights, and key takeaways from a bilateral meeting held at the French Embassy in Brasília in November 2024, titled “Antimicrobial Resistance: How to Change the Game by 2050.” The meeting brought together specialists, scientific societies, policymakers, and representatives from leading academic and institutional organizations from Brazil, France, the United States, and the United Kingdom, fostering essential dialogue on surveillance advancements, diagnostic innovation, and global cooperation (29). During the meeting, Professor Dr. Carlos Kiffer, from the Special Laboratory of Clinical Microbiology at UNIFESP – Brazil and vigiRAM Project (Strengthening a Brazilian Antimicrobial Resistance Surveillance System, https://www.vigiram.org.br), expressed his enthusiasm in discussing the perspective of Brazilian national public health authorities. He highlighted the interface between applied research activities and AMR data in large territories such as Brazil, and even at the global level, noting that “we live in an era of abundant data but limited information” (see Supplementary material).

In this context, this review evaluates Brazil’s current policies and practices in diagnostics, stewardship, and surveillance to identify strengths, gaps, and opportunities for improvement, ultimately supporting evidence-based recommendations to strengthen the national and regional response to AMR. Specifically, it examines Brazil’s alignment with global frameworks such as the WHO Global Action Plan and the One Health approach, the main structural and operational barriers that hinder progress, and the context-sensitive strategies needed to enhance preparedness and control efforts in the coming decade.

## 2 Methodology

This manuscript is a narrative policy-and-practice review. This methodology was intentionally selected instead of a systematic review because the objective is not to address a single, narrowly scoped research question. Narrative reviews are better suited for synthesizing and interpreting broad, complex, and heterogeneous bodies of evidence, enabling conceptual critique, identification of structural gaps, and the integration of diverse insights relevant to multifaceted public health challenges.

This integrative methodology provides a more comprehensive and contextually grounded understanding of the misalignments between policy design and real-world implementation in Brazil’s AMR response. Although it departs from the structure of conventional systematic reviews, this approach is both intentional and methodologically appropriate. By drawing on sources beyond academic publications—such as institutional documents, operational guidelines, and surveillance dashboards—it captures essential implementation-level information typically absent from the scientific literature.

This type of synthesis is particularly valuable in settings where stewardship practices, policy execution, and health-system performance are insufficiently documented through formal research channels but are crucial for understanding how national AMR strategies unfold in practice. Consequently, the methodology enhances ecological validity and yields a more accurate depiction of systemic barriers, operational gaps, and contextual determinants shaping AMR control in Brazil. These insights, in turn, support more informed policymaking, guide resource allocation, and help align future interventions with on-the-ground realities.

To build this synthesis, we adopted a best-evidence approach integrating three complementary evidence streams and covering the period from 2000 to 2025. Inclusion criteria comprised documents addressing antimicrobial resistance, stewardship, diagnostics, surveillance, or AMR-related policy in Brazil or Latin America; publications in English or Portuguese; and materials offering empirical data, policy analyses, or expert perspectives. Exclusion criteria included studies lacking direct relevance to AMR, those focused exclusively on basic laboratory research without policy or clinical implications, editorials without analytical content, and duplicated sources. The first evidence stream consisted of primary evidence from peer-reviewed literature identified through a non-systematic search of PubMed, SciELO, and Google Scholar using terms such as “antimicrobial resistance,” “Brazil,” “Latin America,” “antimicrobial stewardship,” “diagnostic stewardship,” “surveillance,” “PAN-BR,” and “One Health.” The second stream comprised analytical and policy-derived evidence from grey literature—national reports, technical guidelines, legislative documents, and survey data—issued by the Brazilian Health Regulatory Agency (ANVISA), the Ministry of Health (MoH), and regional or international bodies including PAHO and WHO. The third stream incorporated expert opinion and policymaker insights gathered during the high-level bilateral meeting “Antimicrobial Resistance: How to Change the Game by 2050,” held at the French Embassy in Brasília in November 2024, as detailed in the introduction and Supplementary material.

## 3 Assessment of policy/guidelines options and implications

This section examines the critical components of the AMR response, including diagnostic stewardship (DS), antimicrobial stewardship (AMS), and surveillance systems, analyzing the current state, policies, challenges, and opportunities for improvement within Brazil.

### 3.1 Diagnostic stewardship in Latin America and Brazil

#### 3.1.1 The foundational role of DS in combating AMR

DS is an essential, coordinated intervention designed to optimize the use of diagnostic tests, guiding therapeutic decisions and thereby improving patient outcomes and enhancing the efficiency of healthcare delivery. In the context of AMR, DS plays a pivotal role by ensuring the appropriate, timely, and effective use of laboratory tests to accurately identify pathogens and their susceptibility patterns, which is fundamental for selecting the correct antimicrobial therapy and minimizing the unnecessary use of broad-spectrum agents. Historically viewed primarily as a laboratory function, DS has evolved into a crucial multidisciplinary effort that requires active collaboration between clinicians, microbiologists, pharmacists, infection prevention specialists, nurses, and hospital administrators. Its core principle is to ensure that the right test is ordered for the right patient at the right time, and that the results are interpreted correctly and acted upon appropriately to guide patient management (30–32). This term was first used by Morgan et al. (33), but has been practiced empirically for many decades ago, at clinical microbiology labs (34).

#### 3.1.2 Strategies for optimizing diagnostic testing

Effective DS programs employ a range of strategies tailored to institutional needs and resources. Key components include the continuous education for healthcare professionals, which is vital to improving their understanding of test indications, limitations, appropriate specimen collection, and result interpretation. This includes educating them on the pitfalls of inappropriate testing, such as false positives or the detection of colonization rather than infection (35).

Also, developing and implementing evidence-based institutional guidelines and diagnostic algorithms help standardize test ordering practices. These protocols can guide clinicians on when specific tests (e.g., blood cultures, procalcitonin, rapid molecular tests) are indicated or discouraged based on clinical presentation and pre-test probability (36).

On the other hand, the adoption of rapid diagnostic tests (RDTs), including molecular assays (like multiplex PCR for pathogen identification and resistance markers), matrix-assisted laser desorption/ionization time-of-flight mass spectrometry (MALDI-TOF MS) for rapid organism identification, and biomarker tests (e.g., procalcitonin to differentiate bacterial from viral infections or guide antibiotic duration), can significantly shorten the time to targeted therapy. Point-of-care tests (POCTs) such as immunochromatography to detect resistance genes also offer potential for rapid results in settings closer to the patient, although their implementation requires careful validation and quality control (32, 37, 38).

Strong communication channels between the clinical microbiology laboratory and prescribing clinicians are essential. This includes timely reporting of critical results, providing interpretive comments on reports, and facilitating consultations to discuss complex cases or unusual findings (39). Laboratories can also implement strategies to report only clinically relevant results or suppress results that might lead to inappropriate treatment (e.g., reporting urine culture results only when significant pyuria is present) (40).

Regularly monitoring diagnostic test ordering patterns and providing structured feedback to individual clinicians or clinical units can help identify areas for improvement and reinforce adherence to guidelines (41).

Finally, utilizing clinical decision support systems (CDSS) integrated within electronic health records (EHRs) can prompt clinicians regarding appropriate test orders, provide alerts for redundant testing, or guide interpretation based on predefined algorithms (42–45).

**Figure 2:**
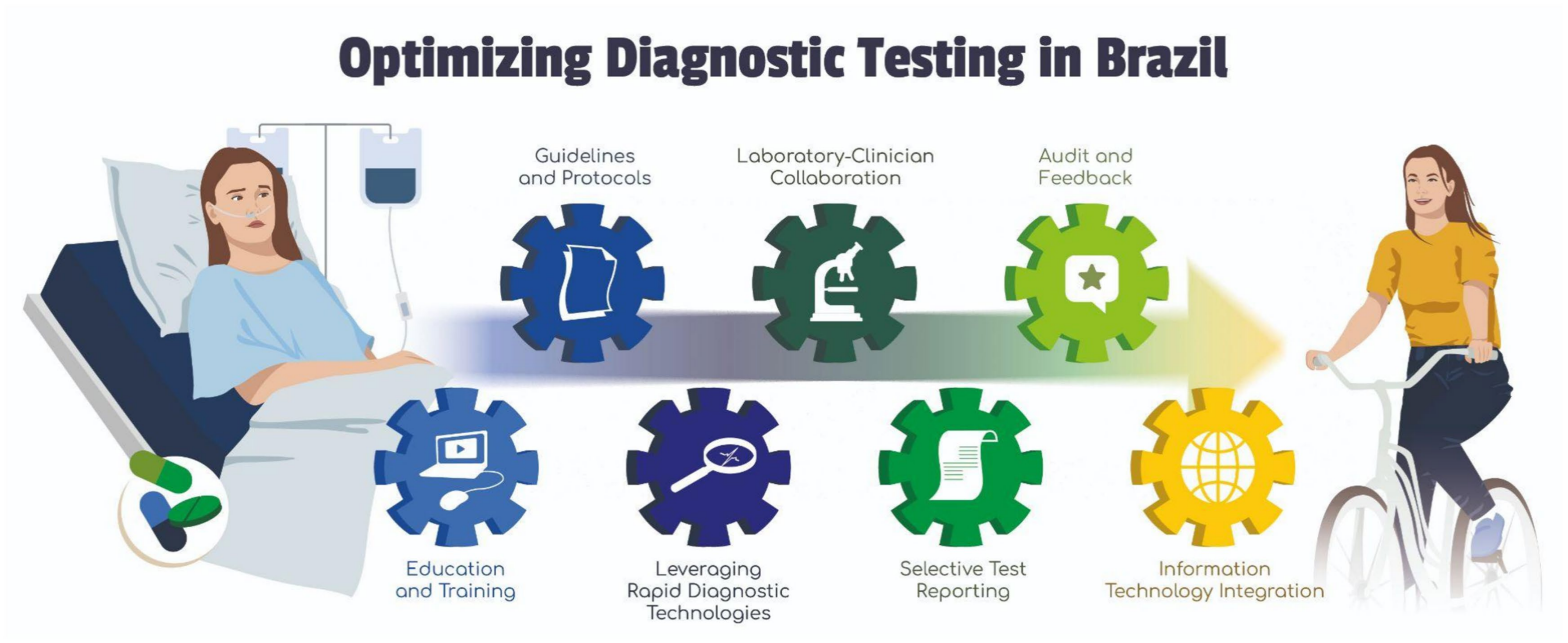
The foundational role of DS in combating AMR. All the elements cited above should work as a sequential gear, where every part propels the engine from disease toward health. Continuous education and training are essential to this process, and they should be provided by public authorities such as MoH and ANVISA (vigiRAM website, webinars, and others). Guidelines and protocols are continuously published by MoH, ANVISA, and BrCAST, such as technical notes, epidemiological alerts, and laboratory guidelines, but also by other academic and scientific societies. One outstanding example is the yearly review of AST-related protocols published by BrCAST, which is managed by four laboratorian professional societies (SBAC, SBI, SBM, and SBPC) with the participation of MoH and ANVISA members. The leveraging of RDTs has been constantly stimulated by CGLAB at a national level within SISLAB (National Public Health Lab System), but also independently as public and private laboratories perceive its cost–benefit advantage. Many Key Opinion Leaders in Brazil have been working to strengthen Laboratory-Clinician Collaboration: most of the national protocols, technical notes, national action plans, and other official documents on AMR, ASP, and DS have been written and reviewed by clinicians (e.g., ID Professionals) and clinical microbiology laboratory experts in partnership with MoH and ANVISA technicians. Selective Test Reporting was once rare in Brazil but is nowadays adopted by many large hospitals across the country. On the other side, many laboratories in Brazil have a long history of adopting external and internal quality control programs, so they are used to constant Audits and Feedback. Finally, there are plenty of examples of Information Technology Integration, especially in large public hospitals, such as preliminary reports of MALDI-TOF results to clinicians, the use of apps and APIs to increase the precision and speed of antimicrobial prescription, and platforms (APIs, apps, software, and systems) that help ID professionals to analyze and critique prescriptions according to the ASP at their hospitals. Illustrations are original works created by MIND (https://www.mind.net.br/).

The coordinated strategies for optimizing diagnostic testing that builds up a DS program and its details are shown in Figure 2. In Brazil, strengthening the response to AMR depends on the integration of continuous education, updated protocols, and close collaboration among clinicians, microbiologists, health authorities, and laboratories. Finally, the incorporation of information technologies has accelerated diagnostics and improved therapeutic decision-making in hospitals.

#### 3.1.3 DS implementation in Brazil: progress and hurdles

While the importance of DS is increasingly recognized, its implementation across Brazil and LATAM faces significant barriers. Limited financial resources restrict investment in essential laboratory infrastructure and advanced diagnostic technologies, particularly within the public sector and in rural areas. Shortages of trained personnel, including clinical microbiologists and laboratory technicians skilled in newer methodologies, further compound the issue. The lack of comprehensive national DS policies and standardized guidelines across different healthcare systems (public vs. private) hinders consistent adoption. There is a need for more evidence assessing the cost-effectiveness of tools that enable faster and more accurate diagnosis in all real-world settings, taking into account the entire patient journey, to determine the added value of these new technologies (19, 46). A recent network meta-analysis found that RDTs combined with an ASP (DSP + ASP) reduced the time to optimal therapy by 29 h (95% CI, −35 to −23 h) compared to blood culture (BC) alone and was associated with a survival benefit (47). Conitec—The National Commission for the Incorporation of Technologies into the Unified Health System—is an expert commission that is continuously evaluating the implementation of new technologies in the nation-wide Brazilian public health insurance program—SUS. Recently, it was approved to use the Multiplex PCR test for detection of multiple bacterial, viral and fungal agents that cause meningitis and encephalitis (48).

Despite these challenges, progress is being made. Brazil’s National Action Plan on AMR includes provisions for enhancing microbiological diagnostics and piloting decision support tools. Several institutions, particularly larger tertiary care centers, have successfully implemented DS initiatives, often integrating them with existing AMS Programs. Studies from the region have demonstrated the positive impact of incorporating RDTs coupled with stewardship interventions, leading to reductions in unnecessary antibiotic use, shorter hospital stays, and improved clinical outcomes. However, these successes are often localized and highlight the need for scalable strategies applicable across diverse healthcare settings. This perception is reinforced by inadequate reimbursement models for laboratory services, which fail to capture the downstream value of an accurate, rapid diagnosis (49).

It is also noteworthy that the concept and practice of AMS appear more widely disseminated and published in the region compared to DS, suggesting a need to elevate the profile and understanding of DS’s critical role (23, 50, 51).

In addition to these known challenges, several structural limitations further hinder widespread DS adoption. Cost-related barriers disproportionately affect under-resourced regions, amplifying disparities between urban centers with advanced diagnostic capability and rural or remote areas where basic microbiology is often unavailable. Moreover, the absence of systematic data on the workforce—particularly regarding training needs, certification gaps, and retention of skilled laboratory personnel—limits the development of targeted capacity-building strategies. These gaps illustrate that strengthening DS in Brazil will require not only technological investment, but also coordinated national policies that address inequities, ensure sustainable financing, and build a competent and adequately distributed diagnostic workforce. We understand that addressing all this challenges is beyond the scope of this review and probably are being discussed in the context of the Brazilan National Action Plan.

#### 3.1.4 The synergistic role of AI in DS and AMS

Artificial intelligence (AI) and machine learning (ML) present transformative opportunities for enhancing both DS and AMS. AI algorithms can analyze vast datasets (clinical, laboratory, epidemiological) to predict the likelihood of infection, identify patients at high risk of resistant pathogens, suggest optimal diagnostic tests, and guide antimicrobial selection. AI-powered CDSS (also called AI-DSS) can provide real-time, patient-specific recommendations integrated into clinical workflows. Furthermore, AI can bolster surveillance efforts by identifying emerging resistance patterns or potential outbreaks much earlier than traditional methods allow. Systematic reviews confirm the high predictive performance of AI tools in AMS/DS contexts. Aligning the development and implementation of these AI tools with regional capacity-building efforts supported by organizations like WHO and Pan-American health organization (PAHO) could provide powerful, scalable solutions to combat AMR in LATAM (43–45, 52–57).

#### 3.1.5 Integrating DS and AMS

Optimizing antimicrobial use requires a tightly integrated approach combining both DS and AMS. Effective DS provides the crucial diagnostic information needed for AMS interventions like de-escalation, targeted therapy based on pharmacokinetic and pharmacodynamic properties, and appropriate treatment duration. Prioritizing policies that foster this integration, investing in both diagnostic technology and stewardship expertise, and promoting continuous education are essential steps for Brazil and LATAM to strengthen their AMR response and preserve antimicrobial effectiveness (38, 56, 58, 59).

### 3.2 AMS in Brazil

#### 3.2.1 The imperative for AMS

AMS encompasses a coordinated set of strategies and interventions designed to optimize the use of antimicrobial agents in healthcare settings (60). The primary goals of AMS are to improve patient outcomes (e.g., cure rates, reduced toxicity), minimize the unintended consequences of antimicrobial use (including the emergence and spread of AMR and *Clostridioides difficile* infections), and ensure cost-effective therapy (61).

Optimizing antibiotic use involves ensuring the selection of the appropriate drug, dose, route of administration, and duration of therapy, tailored to the specific pathogen and site of infection, based on the best available clinical and microbiological evidence. Given the global crisis of escalating AMR, robust AMS programs are no longer optional but a fundamental requirement for safe and effective healthcare delivery (62, 63).

**Figure 3:**
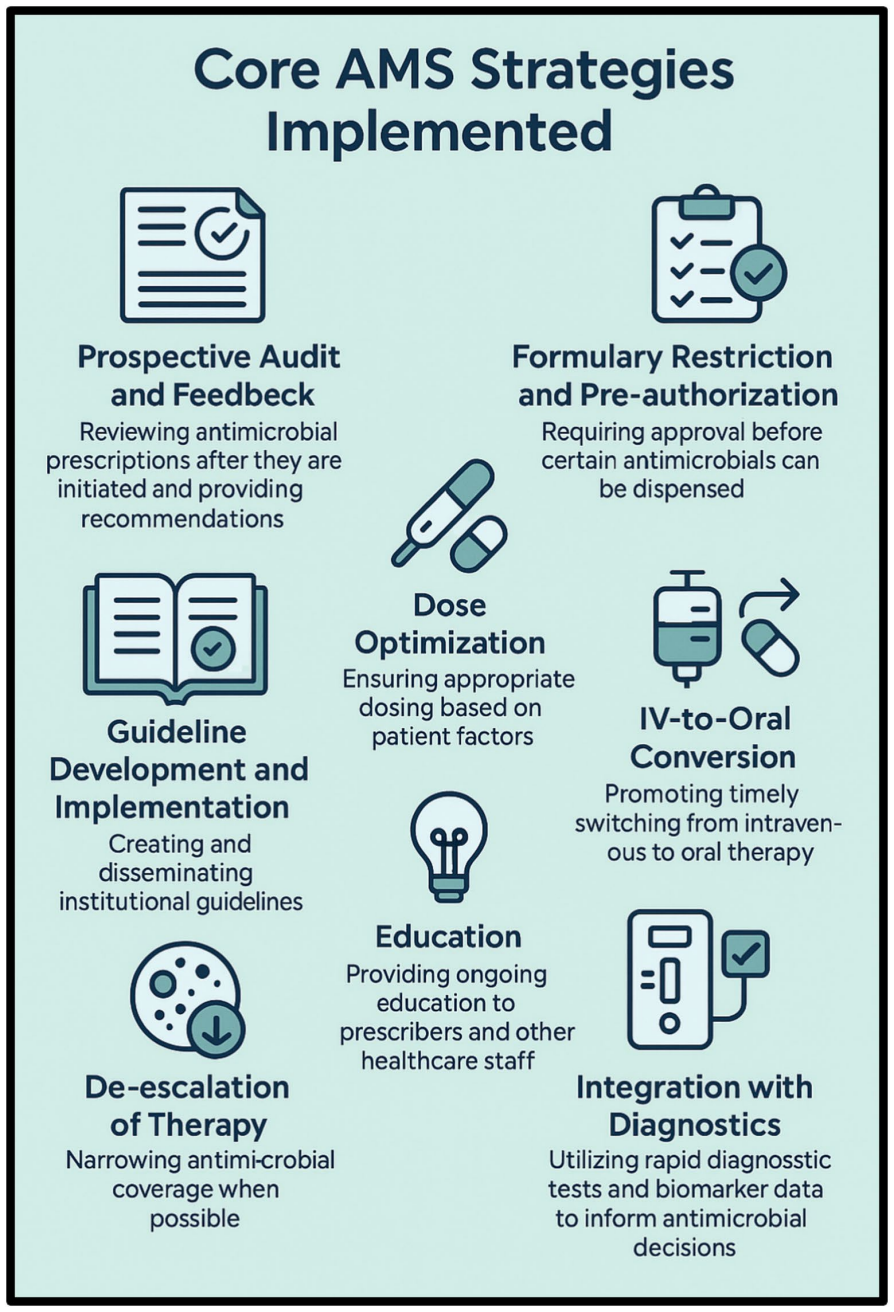
Mapping core elements of AMS in Brazil: from strategy to outcomes. The elements outlined above serve as a conceptual framework to support the multidisciplinary team in visualizing the core components of antimicrobial stewardship program (ASP), aligned with a more strategic and evidence-based approach. These key strategies must be systematically measured and structured to generate actionable outcomes that activate subsequent processes, thereby optimizing resource allocation and contributing to the mitigation of antimicrobial resistance. Illustrations are original works created by MIND (https://www.mind.net.br/).

However, effective implementation of AMS requires institutional leadership, multidisciplinary support with a critical role played by clinical pharmacists, continuous monitoring of antimicrobial use, performance indicator analysis, and ongoing education of healthcare teams (64). A general overview of the main strategies needed to implement an antimicrobial stewardship program is given in Figure 3. The elements described in the figure guide a more strategic, structured, and evidence-based approach, allowing key interventions to be continuously planned, implemented, and evaluated. These strategies must be systematically measured, monitored, and organized to provide concrete and actionable results. In this way, outcomes are strengthened through more efficient allocation of available resources and contribute directly to reducing and controlling antimicrobial resistance. The strengths and weaknesses of the process must be addressed both locally and across the entire healthcare institution, with the engagement and persuasion of healthcare professionals and the families directly and indirectly involved.

While the recommendations outlined align with international best evidence, several require substantial fiscal and administrative capacity that may not be immediately feasible across all Brazilian healthcare settings. Therefore, a structured feasibility analysis—combined with a phased implementation plan prioritizing short-, medium-, and long-term actions according to available resources and existing infrastructure—would enhance the practicality and sustainability of AMS expansion in Brazil, ensuring that efforts are appropriately sequenced and achievable within the country’s diverse healthcare landscape.

#### 3.2.2 The AMR landscape and drivers in Brazil and LATAM

Latin America faces a particularly high burden of AMR. Factors contributing to this include high rates of antimicrobial consumption (often driven by inappropriate prescribing and easy access), inadequate regulatory oversight in some areas, limited access to diagnostics, leading to empirical broad-spectrum use, and insufficient healthcare infrastructure, particularly for infection prevention and control (IPC) (65, 66).

Consequently, the prevalence of MDROs is alarmingly high. Studies consistently report widespread resistance among key pathogens, including carbapenem-resistant Enterobacterales (CRE, especially KPC and NDM-producing *K. pneumoniae*), carbapenem-resistant *A. baumannii* complex (CRAB), extended-spectrum beta-lactamase (ESBL)-producing *Escherichia coli*, and methicillin-resistant *Staphylococcus aureus* (MRSA) (8, 15, 16, 22). The impact of this resistance is significant, contributing to increased patient morbidity, mortality, and substantial healthcare costs. The non-prescription availability of antibiotics in some countries further fuels misuse and resistance development, making structured AMS interventions critically important (67–71).

#### 3.2.3 AMS progress and initiatives in LATAM and Brazil

Despite the challenges, considerable efforts are underway across LATAM to implement and strengthen AMS programs. These initiatives are often driven by national policies, international collaborations, and dedicated healthcare professionals.

- Regulatory Frameworks: Several countries have tightened controls over antibiotic sales. Brazil stands out with ANVISA’s 2010 mandate requiring prescriptions for all antibiotics, significantly reducing over-the-counter access. Other nations like Argentina, Chile, and Colombia have also implemented stricter regulations (72).
- National Action Plans (NAPs): Following the WHO’s call for NAPs, many LATAM countries have developed strategic plans. Brazil launched its first NAP (PAN-BR) for 2018–2022 and is preparing an updated version for 2025–2030. These plans typically emphasize surveillance, stewardship, IPC, research, and One Health approaches. Notably, 87% of PAHO member countries have established NAPs, exceeding the global average (23)
- Surveillance Networks: Regional networks like ReLAVRA (supported by PAHO) (20), national systems like GAL, BR-GLASS and vigiRAM in Brazil are crucial for providing the epidemiological data needed to inform stewardship guidelines and interventions (22, 25).
- Hospital-based AMS Programs: Many larger hospitals, particularly tertiary care and academic centers, have established formal AMS programs. These often involve multidisciplinary teams (infectious disease physicians, clinical pharmacists, nurses, and microbiologists) and implement core stewardship strategies like formulary restriction, pre-authorization, prospective audit with feedback, and guideline development. ANVISA’s “Stewardship Brasil” project conducted national surveys (in 2019, 2022, 2024) to assess AMS implementation in ICUs (24, 62, 73, 74). The 2019 ANVISA “Stewardship Brasil” project, a prospective cross-sectional study of 954 hospitals with adult ICUs (representing 25,565 beds across all 27 states), found that only 47.5% (453) had an established ASP (24). On the other hand the IMPACTO-MR *program,* financed by the federal government initiative called PROADI-SUS (a MoH tax incentive program in partnership with world-class Brazilian hospitals), compiled data from more than 50 Hospitals ICUs throughout Brazil.
- Collaborative Initiatives: Groups like the Brazilian Collaborative on antimicrobial stewardship bring together professionals from different societies, for example, the Brazilian Association of Professionals in Infection Control and Hospital Epidemiology (ABIH), to unify debates and leverage actions. International projects, such as the PAHO-EU collaboration involving Brazil and other Latin American countries, aim to support NAP implementation (19, 58, 64, 75).
- Education and Awareness: Training programs for healthcare professionals (e.g., Brazil’s vigiRAM focusing on microbiology and infectious diseases, https://www.vigiram.org.br) (76) and public awareness campaigns (like World Antimicrobial Awareness Week) are employed to promote responsible antimicrobial use. Incorporating AMS principles into medical and pharmacy curricula and other health-related fields has proven to be a fundamental strategy to prepare future professionals to face the growing challenge of AMR. Teaching concepts such as rational use of antimicrobials, pharmacokinetics and pharmacodynamics, microbiological surveillance, interpretation of laboratory tests, and evidence-based prescribing practices from the undergraduate level helps to train more aware and technically prepared professionals. Moreover, integrating AMS into educational programs promotes a necessary cultural shift in antimicrobial use, encouraging responsible attitudes from the very beginning of training. Universities in several countries—including Brazil—have already started implementing courses, modules, and practical activities focused on AMS, often in collaboration with teaching hospitals and residency programs. This multidisciplinary and educational approach is essential to strengthen public health policies and sustain the effectiveness of antimicrobials in the long term (32, 76–79).

#### 3.2.4 Challenges to AMS implementation in Brazil and LATAM

Despite progress, significant hurdles remain, such as resource limitations, inconsistent implementation and adherence and others. The main challenges are represented in Figure 3.

#### 3.2.5 Enhancing AMS: a multifaceted approach

Moving forward requires a comprehensive strategy. This strategy includes strengthening national policies and commitment by enforcing regulations, allocating dedicated funding, and ensuring sustained political will. It also involves expanding AMS programs beyond hospitals to primary care, outpatient settings, and long-term care facilities (80).

Furthermore, improving surveillance and diagnostics through investment in laboratory capacity, data infrastructure, and rapid diagnostics is crucial to provide timely information for stewardship decisions (34, 81). Promoting multidisciplinary teams and collaboration, fostering teamwork among healthcare professionals, administrators, and policymakers under a One Health umbrella is also essential (82). Investing in education and awareness through continuous professional development and sustained public campaigns is vital (83). Leveraging technology, such as EHRs, CDSS, telehealth, and AI/ML tools, can support prescribing and monitoring (44, 84, 85). Finally, fostering research and innovation to support research on AMS effectiveness, new diagnostics, alternative therapies, and behavioral interventions will be key to long-term success.

By addressing these priorities through a collaborative, interdisciplinary, and sustained approach, Brazil and LATAM can significantly advance their AMS efforts, preserving the effectiveness of existing antimicrobials and mitigating the profound threat of AMR.

### 3.3 Strengthening surveillance systems

Despite these important advances, Brazil’s surveillance architecture still faces critical limitations that constrain its overall effectiveness. Coverage remains uneven across regions, with underrepresentation of low-resource, rural, and Northern states, creating geographic blind spots that limit the generalizability of national estimates. Participation by private hospitals, responsible for a significant proportion of complex care, is also inconsistent, leading to potential selection bias toward public tertiary centers. Furthermore, most surveillance platforms rely on sentinel sites and convenience sampling rather than population-based methodologies, restricting the capacity to infer national incidence or burden of AMR. Data quality varies widely, reflecting heterogeneity in laboratory capacity, AST methodologies, and reporting completeness, while outcome data (e.g., mortality, length of stay, costs) remain largely absent outside specialized initiatives such as IMPACTO-MR. Significant delays in data integration across systems (BR-GLASS, ANVISA, GAL) hinder real-time threat detection. Together, these gaps underscore the need for investments in harmonization, representativeness, and analytics to ensure that Brazil’s surveillance system not only generates high volumes of data but produces reliable, interpretable, and actionable information for clinical decision-making and policy planning.

#### 3.3.1 The cornerstone of AMR control

**Figure 4:**
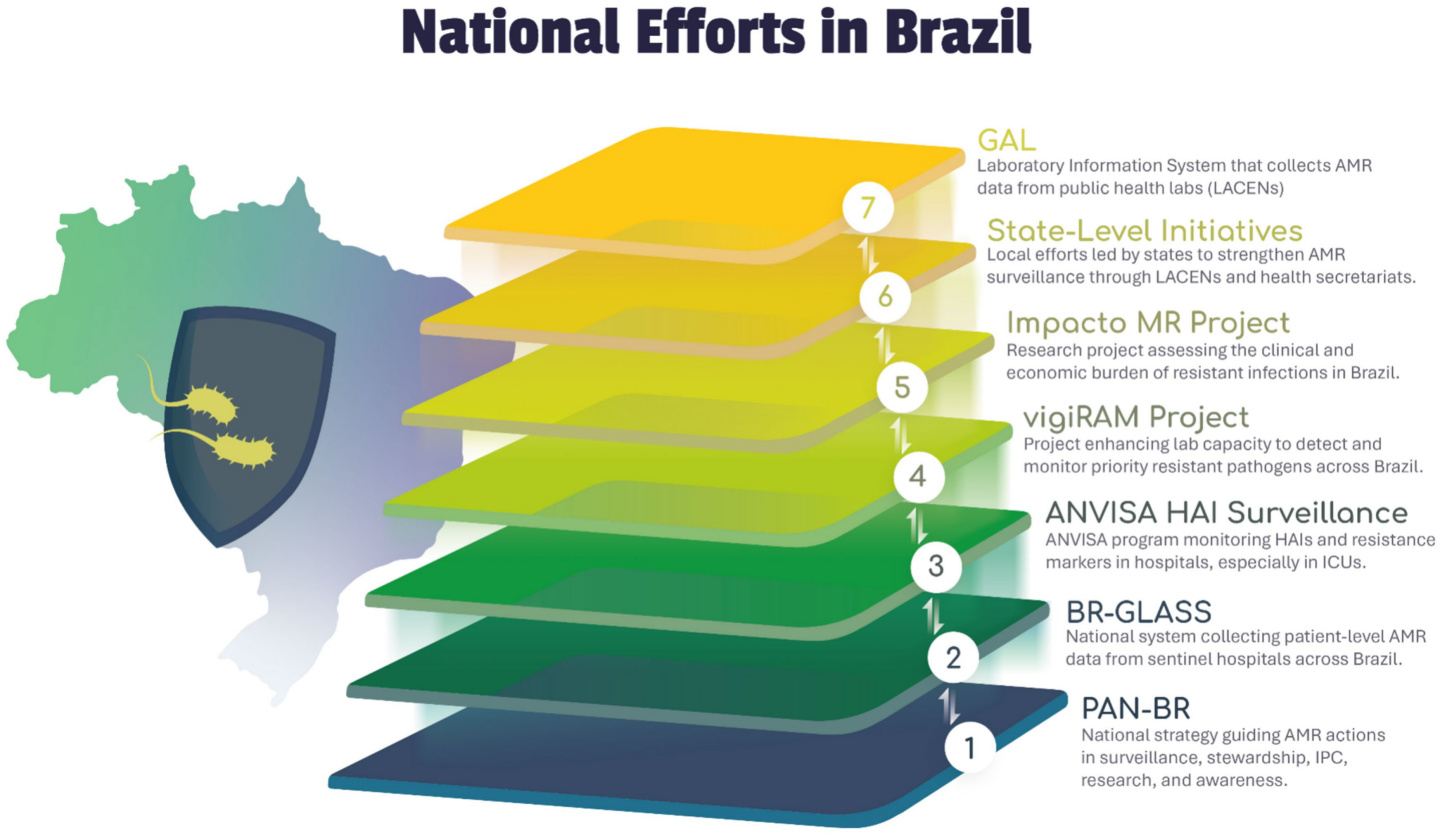
Efforts in Brazil to implement AMR surveillance. This figure illustrates Brazil’s integrated national strategy for combating antimicrobial resistance (AMR), re-conceptualized with the foundational national policy at its base. The pyramid builds upward from the overarching strategic plan through layers of national surveillance, targeted research, and operational capacity-building, culminating in the specific laboratory information systems that capture data at the ground level. Each layer performs a distinct function while interfacing with the others to create a cohesive public health response aligned with the “One Health” approach. 1—PAN-BR: National “One Health” strategic action plan for AMR. 2—BR-GLASS: National sentinel surveillance system aligned with WHO-GLASS. 3—ANVISA HAI Surveillance: Mandatory national surveillance of HAIs and resistance in ICUs. 4—vigiRAM Project. 5—Impacto MR Project: a SUS-funded research platform, that researches the clinical and economic burden of resistant infections in ICUs. 6—State-Level Initiatives: State-level public health labs (LACENs) that validate and analyze isolates. 7—GAL: National public health laboratory information system for standardized data entry and management. Illustrations are original works created by MIND (https://www.mind.net.br/).

Robust surveillance of AMR and antimicrobial use (AMU) is the bedrock upon which effective control strategies are built. Timely, accurate, and representative surveillance data are essential to understand the magnitude of the AMR problem, monitor trends over time and geography, detect emerging threats, inform clinical guidelines, guide stewardship interventions, evaluate the impact of policies, and advocate for necessary resources. Brazil, as well as other Latin American countries participate in and contribute to a multi-tiered surveillance architecture, encompassing global, regional, and national systems (see Figure 4).

#### 3.3.2 Global framework: WHO GLASS

At the apex is the World Health Organization’s Global Antimicrobial Resistance and Use Surveillance System (GLASS), launched in 2015. GLASS aims to standardize the collection, analysis, and sharing of AMR and AMU data globally. It provides a framework and methodology for countries to build or strengthen their national surveillance systems. Key components include surveillance of AMR in common human bacterial pathogens (priority pathogens like *E. coli, K. pneumoniae, A. baumannii, P. aeruginosa, S. aureus, Streptococcus pneumoniae, Salmonella* spp., and *Neisseria gonorrhoeae*) isolated from clinical specimens (blood, urine, stool, genital swabs), and monitoring of antimicrobial consumption (86). Brazil officially joined GLASS in 2017 and began submitting data, demonstrating its commitment to global standards and data comparability (22).

#### 3.3.3 Regional collaboration: PAHO/ReLAVRA

The Pan American Health Organization (PAHO) coordinates the Latin American Antimicrobial Resistance Surveillance Network (Red Latinoamericana de Vigilancia de la Resistencia a los Antimicrobianos, ReLAVRA). Established years before GLASS, ReLAVRA has been instrumental in building laboratory capacity and promoting standardized AMR surveillance across the region. It aggregates data annually from national reference laboratories in 20 participating countries, providing a crucial regional overview of resistance patterns. Between 2000 and 2014, the network analyzed data from over 2.6 million isolates (20).

#### 3.3.4 National efforts in Brazil

Brazil has developed a multi-pronged national surveillance strategy, integrated within its broader AMR action plan, acting in different levels that are being articulated to complement each other, in its different levels, such as BR-GLASS, ANVISA-HAI dashboard, vigiRAM, and GAL. Figure 4 represents a conceptual reorganization of the Brazilian national strategy for addressing AMR, structured around a national policy that provides the normative and strategic framework for all subsequent actions. The pyramid develops upward, beginning with the national strategic plan that defines priorities and guidelines, followed by the epidemiological surveillance systems that monitor the occurrence and trends of AMR in the country. At higher levels, the framework incorporates components of specific surveillances and research systems, as well as operational capacity building, both essential for strengthening the technical response of healthcare services. The apex of the structure consists of specialized laboratory systems responsible for capturing, processing, and integrating microbiological data derived from reference centers. Each layer performs specific and complementary functions, creating functional interfaces that enable a coordinated, consistent, and evidence-based response, thereby reinforcing the principles of the One Health approach.

- PAN-BR: The foundation of Brazil’s entire AMR framework is the National Action Plan for the Prevention and Control of Antimicrobial Resistance (PAN-BR). Established in 2018, this comprehensive, multi-sectoral strategy aligns with the WHO’s Global Action Plan and fully embraces the “One Health” approach, integrating human, animal, and environmental health sectors. Coordinated by the Ministry of Health with other key agencies like the Ministry of Agriculture (MAPA) and the Brazilian Health Regulatory Agency (ANVISA), PAN-BR sets the five core national objectives: improving awareness, strengthening surveillance, reducing infection incidence, optimizing antimicrobial use, and developing the economic case for sustainable investment (25).
- BR-GLASS: Brazil’s national surveillance program (BR-GLASS) was built upon a successful pilot. The 2018 pilot study in Paraná state was substantial, compiling results from 11,347 isolates from just three hospitals (22). This system is now mature and integrated with other national reporting structures, such as vigiRAM and peaked 40,846 isolates, from 19 states, in 2024 (Pillonetto M, personal communication, www.vigiram.org.br). This demonstrated the system’s capacity for high-volume, high-quality data collection, and consistent contribution to global data through WHO-GLASS program (87, 88).
- ANVISA’s national surveillance program for Healthcare-Associated Infections (HAIs) runs parallel to BR-GLASS. Brazilian hospitals with ICU beds must report monthly data on key infections (e.g., central line-associated bloodstream infections, ventilator-associated pneumonia) and their pathogens’ resistance profiles. This system offers nationwide, continuous AMR trend data from critical healthcare settings, crucial for regulatory oversight and identifying public health threats. ANVISA’s surveillance network, which also feeds into this effort, currently receives data from 3,486 hospitals with ICU beds (85).
- vigiRAM project: The vigiRAM project is a key intervention funded by the U.S. CDC to *solve* the harmonization challenge. It strengthens the BR-GLASS network by providing training, standardizing protocols, and enhancing quality control. In one initiative, it trained 218 participants at 42 laboratories in 25 of Brazil’s 27 states. The project’s surveillance component has produced critical, actionable data, showing the rapid increase in *bla*_NDM_ genes detection in blood cultures form ICUs. Also it showed a 39 h decrease in carbapenemase production, with the use of RDT, that could have a huge impact in HAI’s costs, and patients outcomes (73).
- IMPACTO-MR Project: As mentioned previously, this PROADI-SUS platform functions as a deep-dive, clinical-economic surveillance tool for Brazilian ICUs. It complements the broader, isolate-based surveillance of BR-GLASS and vigiRAM by providing patient outcome and cost data on MDROs from over 50 ICUs.
- State-Level Initiatives: LACENs are state-level reference laboratories forming the operational core of Brazil’s national public health network. They receive microbial isolates from hospitals for confirmatory testing, quality control, and advanced resistance mechanism characterization. Crucially, LACENs validate local data before it enters national systems, ensuring data accuracy and reliability for national trends and policies, through GAL National Database.
- GAL National Database: The *Gerenciador de Ambiente Laboratorial* (GAL) is a nationwide, computerized laboratory information system (LIS) developed by DATASUS and CGLAB. It standardizes workflows and data entry for the National Network of Public Health Laboratories (SISLAB). GAL is the official system for registering microbial isolates and antimicrobial susceptibility test results for AMR surveillance, aggregating local data into a national database for analysis. The Ministry of Health is actively using this database to strengthen AMR surveillance. A 7-year (2015–2022) surveillance analysis of more than 83,000 isolates tested for *bla*_KPC_ and *bla*_NDM_ demonstrated a significant 41.1% annual percent increase in *bla*_NDM_ in Enterobacterales, while *bla*_KPC_ is slightly declining. This finding has direct, urgent implications for empirical therapy guidelines across the country (8).

##### 3.3.4.1 Monitoring antimicrobial use (AMU)

A historic weakness in Brazil’s surveillance (see W3, Table 1) has been the lack of a robust, integrated national system for monitoring antimicrobial *consumption*. However, data from the National System for the Management of Controlled Products (SNGPC) is beginning to fill this gap. A 2024 time-series study analyzing SNGPC data found a 30% overall increase in antibiotic consumption in Brazil from 2014 to 2019 (89). This data also proved invaluable for monitoring public health shocks, revealing a dramatic spike in azithromycin consumption during the COVID-19 pandemic, which was often prescribed inappropriately, and a corresponding drop in amoxicillin use (73).

**Table 1:** SWOT analysis of AMR response infrastructure and strategy in Brazil*.

Strengths	Weaknesses
S1. Strong alignment with WHO Global Action Plan on AMR.	W1. Lack of dedicated resources for the PAN-BR: limited resources and funding for full-scale implementation across all sectors.
S2. Comprehensive multisectoral approach integrating human, animal, and environmental health (One Health): skilled professionals in AMR, including infectious disease specialists, nurses, pharmacists, and microbiologists; access to high-income country (HIC) technologies.	W2. Regional disparities in healthcare access and laboratory capacity: absence of national studies on the AMR burden.
S3. Government commitment through a structured national action plan (PAN-BR AMR 2025–2030). Active participation in global AMR governance and policymaking: participation of health managers in global AMR forums (G20, UNGA, WHA).	W3. Limited comprehensive, integrated national antimicrobial consumption (AMU) monitoring system, though SNGPC data is emerging. Challenges in integrating data systems and intersectoral coordination, for strengthening a one-health approach.
S4. Availability of public health infrastructure via the Unified Health System (SUS): 27 reference labs (LACENs), and 3,486 hospitals reporting HAI data.	W4. Need for consistent training and capacity-building among healthcare professionals.

Opportunities	Threats
O1. Revolution in diagnostic microbiology: expansion of international collaborations for research and surveillance.	T1. Inadequate reimbursement for microbiology services: risk of over-reliance on external funding and technical support. Emerging infectious diseases diverting attention and resources.
O2. Advancements in antimicrobial stewardship: development of innovative antimicrobial stewardship programs.	T2. Lack of multidisciplinary teams in IPC; pharmacists and microbiologists not always involved. Decreasing interest among new healthcare professionals in microbiology and IPC fields.
O3. Greater integration of environmental surveillance and climate change response.	T3. Limited strategic vision among hospital administrators—failure to view laboratory services as investments rather than expenses. Socioeconomic and political instability potentially affecting long-term policy continuity
O4. Global awareness and sensitivity to the AMR issue: engagement of civil society and academic institutions to strengthen awareness and implementation.	T4. Escalating the global burden of AMR may outpace national mitigation efforts.

*Based on authors’ knowledge and opinion.

##### 3.3.4.2 Risks, challenges and to dos in surveillance

Despite existing structures, significant challenges continue to impede the effectiveness and comprehensiveness of AMR surveillance in both Brazil and LATAM. A primary issue is ensuring high-quality, complete, and representative data. This is often difficult due to the variability in laboratory capacity, diagnostic methods, quality control practices, and data entry across different institutions and regions, which inevitably leads to inconsistencies and potential biases. Furthermore, achieving truly representative national data is challenging due to undersampling in specific areas or populations, compounded by Brazil’s vast size and regional inequalities, which result in surveillance infrastructure and capacity being concentrated in more developed urban centers, leaving remote or less-resourced areas under-surveilled. A notable gap is Brazil’s current lack of robust national data on overall antimicrobial consumption (25, 89, 90).

To address these critical issues, several enhancements are necessary. It is crucial to expand and enhance national surveillance initiatives, such as BR-GLASS and other national surveillance networks (e.g., ANVISA HAI surveillance), by increasing the number and geographic representativeness of sentinel sites. Alongside this, implementing robust data quality checks and ensuring timely reporting and feedback are essential for improving the accuracy and utility of the collected information (22). A pivotal step would be to establish mandatory AMR reporting, creating a national system for compulsory reporting of critical AMR phenotypes (e.g., carbapenem resistance) and potentially specific resistance mechanisms from all clinical laboratories to public health authorities. These subjects are being actually discussed and planned within the AMR technical group at MoH.

Moreover, developing national AMU monitoring is vital; this involves creating a system for systematically collecting and analyzing antimicrobial consumption data from various sectors, including hospital inpatient, outpatient/community pharmacy, and veterinary, to understand usage patterns and correlate them with resistance trends (8, 9, 20). Investing in health information technology, by promoting the adoption and interoperability of EHRs and Laboratory Information Systems (LIS) capable of supporting surveillance data, would significantly streamline data collection and analysis (32, 34, 44). Nevertheless, resource constraints remain a considerable hurdle, as sustaining and expanding surveillance systems requires continuous investment in laboratories, technology, personnel, training, and data management infrastructure, which can be particularly challenging in resource-constrained settings (13, 45, 62).

#### 3.3.5 Moving forward: recommendations for strengthening surveillance

Addressing these challenges requires a concerted effort. This includes establishing mandatory reporting policies that advocate for mandatory notification of critical AMR phenotypes or genotypes to public health authorities (1, 20, 25). Additionally, continued dialogue and technical work are needed to harmonize antimicrobial susceptibility test (AST) interpretation between BrCAST and CLSI/EUCAST where feasible, or to develop clear methods for data translation to facilitate comparisons. Sustained investment is crucial, securing long-term funding and political commitment for surveillance infrastructure, technology, and personnel training (13, 18, 62). Leveraging technology by exploring the use of AI and data analytics can enhance trend analysis, outbreak detection, and predictive modeling based on surveillance data, to separate viral from bacterial infection or to prescribe antimicrobials when needed (43–45, 52). Finally, strengthening mechanisms for sharing and integrating surveillance data across human, animal, and environmental sectors will enhance One Health Integration. By tackling these challenges, Brazil can solidify its position as a regional leader in AMR surveillance, generating the high-quality data needed to drive effective national policies and contribute meaningfully to global AMR control efforts through ReLAVRA and GLASS (3, 18, 20, 23, 76).

## 4 SWOT analysis of AMR response in Brazil

Here in Table 1, we propose a SWOT (Strengths, Weaknesses, Opportunities and Threats) analysis regarding the scenario of AMR response in Brazil, but that could be useful and adapted to many other countries in the region.

The proposed recommendations are aligned with the actions needed to address AMR and reflect the progress already achieved in Brazil. However, several of these recommendations require substantial fiscal, administrative, and operational capacity, which may limit full implementation in the short term. Therefore, a feasibility analysis is recommended, considering human resources, infrastructure, budget, and institutional maturity. In addition, an incremental implementation plan is suggested, with priorities distributed across short-, medium-, and long-term horizons according to the specific conditions of the Brazilian health system.

In the short term, efforts should focus on consolidating interoperability across information systems (vigiRAM, BRGLASS, SNGPC, SINAN, Notivisa, PNASS, among others). An important opportunity is to prioritize strengthening LACENs and sentinel laboratories as the central axis of national surveillance. Another proposal is to facilitate real-time dissemination of standardized minimum indicators for antimicrobial consumption and use in healthcare services.

To reduce regional disparities in laboratory and healthcare capacity in the short to medium term, it is recommended to map gaps in infrastructure and diagnostic access, implement federal funding targeted to regions with lower operational capacity, and expand tele-microbiology and tele-infectology programs to provide remote clinical and laboratory support. In parallel, it is essential to strengthen training and continuous education in AMR, Stewardship, and IPC by developing training pathways for multiprofessional teams, encouraging the recruitment and development of new specialists in clinical microbiology and IPC, fields that are experiencing declining interest, and integrating these topics into undergraduate and residency curricula. In the medium term, expanding the use of rapid diagnostic technologies and strengthening applied research should be prioritized through cost-effectiveness analyses, international partnerships for technology transfer, and the promotion of studies on the national AMR burden, which currently remain limited in scope. It is also recommended to structure innovative ASP that can be adapted to hospitals of different sizes, enhance communication between clinical and laboratory teams (including the use of tools such as MALDI-TOF and PCR) and broaden access to digital platforms and decision-support systems. Over the medium to long term, environmental surveillance should be expanded by integrating water, soil, and effluent data into AMR monitoring platforms and coordinating actions with environmental regulatory agencies. In the short term, increasing engagement of civil society, academia, and hospital administrators is crucial through national campaigns framing AMR as a public health priority and by promoting the understanding of laboratories as strategic investments rather than cost centers. Finally, a strategic prioritization plan (e.g.: within PAN-BR) based on political, administrative, and budgetary feasibility should be developed, classifying actions by complexity, impact, and cost: in the short term (1–2 years), implement low-cost, high-impact measures such as training, data integration, and standardized indicators; in the medium term (3–5 years), expand infrastructure and incorporate new technologies; and in the long term (5 + years), consolidate robust surveillance systems, integrate environmental monitoring, and achieve full expansion of Stewardship initiatives.

## 5 Actionable recommendations

Building upon the assessment of policies, stewardship programs, surveillance systems, and the SWOT analysis, the following actionable recommendations are proposed to strengthen the fight against AMR in Brazil and LATAM:

### 5.1 Enhancing stewardship (DS and AMS)

To strengthen integrated antimicrobial stewardship programs, national policies should mandate their implementation in all hospitals above a certain size, providing clear guidelines, standardized metrics, and potential financial incentives or support mechanisms, especially for public and smaller facilities, thereby ensuring widespread adoption and effectiveness. Potential barriers include limited institutional resources, insufficient trained personnel, heterogeneous digital infrastructures, and variable leadership engagement. Furthermore, stewardship efforts need to extend beyond hospitals: tailored drug stewardship and AMS interventions should be developed and piloted in primary care clinics, dental practices, and long-term care facilities, taking into account prescribing patterns and the specific challenges of these settings (19, 28). Key barriers involve fragmented outpatient data systems, high provider turnover, and lack of stewardship culture outside hospitals. Indicators may include the percentage of outpatient facilities implementing AMS activities, appropriateness of antimicrobial prescriptions, and reductions in unnecessary broad-spectrum antibiotic use. During her lecture on rising AMR rates in the context of COVID-19, Twisha S. Patel, from the Division of Healthcare Quality Promotion at the U.S. CDC, highlighted that this global health crisis created opportunities to implement and strengthen best practices in AMS, including preparations for future pandemics (see Supplementary material).

Alongside this expansion, significant public and private investment is crucial for strengthening clinical microbiology laboratories, ensuring access to essential conventional diagnostics and antimicrobial susceptibility testing (AST) capacity, aligned with standards like BrCAST/CLSI (30, 62, 81). A phased implementation of rapid diagnostic tests (RDTs), such as MALDI-TOF, molecular assays for resistance markers, and biomarker tests like procalcitonin, should also be prioritized, integrated seamlessly with DS protocols (32, 37).

Moreover, actively promoting and potentially mandating the inclusion of clinical pharmacists and clinical microbiologists as core members of hospital infection prevention and control (IPC) and AMS committees will facilitate truly multidisciplinary decision-making (46). Finally, developing and regularly updating evidence-based national guidelines for the diagnosis and treatment of common infectious syndromes is paramount (26, 68). These guidelines should incorporate local resistance data (national/regional antibiograms) and offer clear recommendations for diagnostic testing and antimicrobial selection/duration, with wide dissemination and integration into clinical practice support tools to ensure their impact (23).

### 5.2 Policy, education, and collaboration

Sustaining political commitment and funding is crucial, therefore, dedicated, long-term funding for the National Action Plan and its associated activities, such as surveillance, stewardship, IPC, and research, must be ensured, moving beyond reliance on existing budgets. Establishing a National Program on AMR, and converging funding sources from different ministries, such as Health, Science and Technology, Agriculture and others would be a game changer. Furthermore, it is vital to advocate for AMR as a standing national health security priority (23).

In addition, exploring and piloting Value-Based Healthcare (VBHC) models related to infection management and AMR control can be highly beneficial, shifting the focus from volume-based reimbursement to rewarding quality outcomes, appropriate diagnostics, and optimized antimicrobial use. This approach could also help address threats related to inadequate reimbursement for microbiology services. In this context, Secretary Maciel emphasized the role of the One Health paradigm in fostering cross-sectoral collaboration (from local to global levels) to tackle complex and emerging challenges such as pandemics, climate change, and antimicrobial resistance. This topic was also highlighted during the embassy meeting in Brasília (see Supplementary material). Strengthening this collaboration involves establishing formal mechanisms for data sharing, coordinated surveillance, and joint interventions between human health, animal health, and environmental sectors. The recent formalization of Brazil’s multi-institutional One Health Committee represents a much-welcomed policy action that now needs to be consolidated (13, 18, 20, 23, 25, 29, 35, 64, 76, 79).

Moreover, enhancing professional training and public awareness is essential. This includes integrating comprehensive AMR, DS, and AMS training into undergraduate and postgraduate curricula for all relevant health professions, as well as implementing sustained public awareness campaigns promoting responsible antibiotic use and hygiene practices. Addressing the declining interest in microbiology and IPC fields is also a key concern (35, 79).

A structured feasibility analysis is essential to guide Brazil’s implementation of AMR policies, given existing constraints in financing, laboratory capacity, workforce availability, and intersectoral coordination. Based on this assessment, a phased implementation plan is recommended. In the short term (1–2 years), priorities should focus on low-cost, high-feasibility actions, including optimizing the use of existing infrastructures, strengthening early cross-sectoral coordination mechanisms, integrating AMR, DS, and AMS content into health professional curricula, and expanding public awareness campaigns, while leveraging participation in regional and global networks to harmonize indicators and accelerate capacity building. In the medium term (3–5 years), efforts should emphasize the establishment of a National AMR Program with convergent funding streams across ministries, piloting value-based healthcare models for infection management, expanding professional training platforms, and strengthening One Health surveillance integration through interoperable data systems and coordinated interventions. In the long term (5 + years), sustained investment is required to consolidate nationwide One Health governance, ensure stable financing for the National Action Plan, reduce structural gaps in human resources through career incentives and advanced training programs, scale effective value-based models, and foster durable international partnerships for diagnostic innovation, technology transfer, and harmonized surveillance. Collectively, this phased approach enables realistic, resource-sensitive implementation while progressively building the infrastructure and institutional maturity needed for a robust national AMR response.

Finally, fostering regional and international collaboration is vital by actively participating in and leveraging regional networks (PAHO, ReLAVRA) and global initiatives (GLASS, G20, ICARS, GLG, Funds) discussions for knowledge exchange, capacity building, joint research, and policy alignment. Strengthening bilateral partnerships, such as Brazil-France, with a focus on specific areas like diagnostic innovation or surveillance harmonization, will further reinforce these efforts (see additional comments on the Supplementary material) (20, 25, 29, 76).

### 5.3 Monitoring and evaluation

To effectively monitor policy interventions and AMS programs, it is crucial to establish clear performance indicators. These should be SMART (Specific, Measurable, Achievable, Relevant, and Time-bound), tracking elements such as appropriate diagnostic testing rates, antimicrobial consumption metrics (e.g., DDDs), adherence to guidelines, prevalence of key MDROs, and coverage of AMS programs. Alongside this, regular audits and feedback loops are essential, involving periodic reviews of stewardship activities, surveillance data quality, and policy implementation at both national and institutional levels, with findings used to refine strategies. Furthermore, developing centralized data platforms is vital for collecting, analyzing, and reporting monitoring data, ensuring accessibility for policymakers and researchers (21, 41, 68, 75–77).

## 6 Discussion

The fight against AMR in Brazil and Latin America is a complex endeavor demanding a sustained and multifaceted response. This review highlights the significant strides made, including the establishment of national action plans, participation in global and regional surveillance networks like GLASS and ReLAVRA, implementation of regulatory controls on antibiotic sales, and the proliferation of hospital-based AMS programs in many centers. Initiatives like BR-GLASS and vigiRAM demonstrate Brazil’s commitment to strengthening national capacity (20, 22, 26, 76).

Furthermore, the growing recognition of the interconnectedness of human, animal, and environmental health, embodied in the One Health approach, as well as engagement in high-level international forums like the G20, signals an increasing political awareness (3, 13, 23). Those topics were deeply discussed and outstanded by qualified speakers, during the bilateral meeting at the French Embassy, such as the lecture by the former Health Surveillance and Environment Secretary, Ethel Maciel, the CDC Stewardship Specialist, Twisha Patel, and also the French government, represented by Pierre-Yves Bello (see Supplementary material) (29). During this meeting, it was concluded that antimicrobial resistance in Latin America constitutes an emerging and highly relevant threat to public health, demanding the implementation of integrated strategies that ensure accurate diagnosis, continuous surveillance, and appropriate therapeutic interventions.

However, profound challenges persist, as illuminated by the SWOT analysis (see Table 1) and the assessment of current policies (see Figures 3, 4). The lack of dedicated funding for national plans, the absence of comprehensive AMR burden studies, incomplete data on national antimicrobial consumption, and geographic disparities in surveillance and healthcare infrastructure remain significant weaknesses. The effective implementation of both DS and AMS is hampered by resource constraints, inconsistent execution across diverse healthcare settings, prevailing cultural and behavioral barriers to optimal prescribing and testing, and a concerning lack of focus on outpatient and primary care settings. Technical challenges, such as the variability in AST interpretation standards and the need for greater integration of advanced diagnostics, further complicate the landscape. Threats such as inadequate reimbursement for essential laboratory services, the frequent absence of truly multidisciplinary teams, and declining interest in relevant specialties risk undermining progress (7, 12, 58, 62).

The critical synergy between DS and AMS cannot be overstated. Effective AMS relies heavily on accurate and timely diagnostic information to guide targeted therapy, enable de-escalation, and avoid unnecessary broad-spectrum use. Conversely, DS initiatives are most impactful when integrated into a broader stewardship framework that ensures diagnostic results translate into appropriate clinical action. Elevating the implementation and integration of DS alongside AMS is paramount. The advent of AI and ML offers significant opportunities to enhance both areas, from predictive diagnostics to optimized prescribing recommendations and improved surveillance analytics, but requires careful validation and equitable implementation (30, 43–45, 52, 57).

These findings align closely with global AMR governance frameworks, reinforcing the core objectives of the WHO Global Action Plan—particularly those related to strengthening surveillance, optimizing antimicrobial use, enhancing IPC, and fostering multisectoral collaboration under One Health. The recommendations also contribute directly to the UN Sustainable Development Goals, including SDG 3 (Good Health and Wellbeing) through improved infection management and reduced AMR-related morbidity and mortality, SDG 6 (Clean Water and Sanitation) through integration of environmental surveillance, and SDG 17 (Partnerships for the Goals) by emphasizing international cooperation and capacity building. Together, these linkages situate Brazil’s AMR efforts within broader global commitments to health security and sustainable development.

### 6.1 Limitations

This review, while aiming for comprehensiveness, has methodological and contextual limitations. Methodologically, the analysis relies predominantly on published literature, publicly available reports, and information derived from a specific bilateral meeting, which may limit the depth of triangulation and exclude unpublished or region-specific data. Additionally, inconsistencies and gaps in available datasets—particularly those related to antimicrobial consumption and the national burden of AMR across One Health sectors—constrain the robustness of comparative analyses. Contextually, Brazil’s large geographic, socioeconomic, and institutional heterogeneity introduces variations that may not be fully captured in this synthesis, and the perspectives presented may reflect the experiences of selected experts and institutions more than the entire national landscape. Moreover, the AMR scenario is rapidly evolving, underscoring the need for continuous reassessment of findings and recommendations to ensure their relevance over time.

### 6.2 Future directions

Looking ahead, several key directions emerge. Firstly, expanding the reach of robust DS and AMS programs beyond tertiary hospitals into primary care, community settings, and long-term care facilities is crucial for tackling the bulk of antimicrobial use. Secondly, strengthening regional and global collaboration through platforms like PAHO, ReLAVRA, and GLASS is essential for harmonizing approaches, sharing best practices, building capacity, and conducting multinational research. Thirdly, the strategic adoption of digital health tools—including interoperable EHRs, telehealth platforms, mobile applications for clinical support, and validated AI/ML algorithms—should be prioritized to enhance surveillance, optimize prescribing, and facilitate stewardship interventions at scale. Fourthly, addressing the pipeline for new antimicrobials, diagnostics, and alternative therapies requires innovative funding models, public-private partnerships, and policies that balance access with stewardship, alongside responsible engagement with the pharmaceutical and diagnostic industries. Finally, reinforcing the One Health approach through concrete intersectoral actions and integrated surveillance systems remains fundamental for a sustainable AMR response. All these tasks would be better performed, checked, implemented and improved with the Guidance of a National Politics Statement on AMR, as well as a National Program on Antimicrobial Resistance Prevention and Control.

## 7 Conclusion

Antimicrobial resistance (AMR) remains an urgent and intensifying threat to health security and economic stability in Brazil and across Latin America, requiring a sustained, coordinated, and adequately resourced national response. This review highlights that strengthening diagnostic stewardship, expanding antimicrobial stewardship programs across all levels of care, enhancing surveillance of resistance and antimicrobial consumption, and advancing coherent, evidence-based policies are fundamental to an effective strategy. The recommendations presented offer a practical roadmap for policymakers and health leaders, centered on technological innovation, multidisciplinary collaboration, equitable access to diagnostics, and long-term political commitment within a One Health framework. Given its scientific capacity, diverse healthcare system, and engagement in regional and global AMR initiatives, Brazil is well positioned to lead regional progress. To catalyze and integrate these efforts, we propose that the Ministry of Health establish a National AMR Program and issue a formal National Policy Statement on AMR, providing the strategic and political foundation necessary to drive sustainable action and safeguard antimicrobial effectiveness for future generations.
