# Supplementary material for: Fighting antimicrobial resistance in Brazil: strengthening diagnostic stewardship, antimicrobial stewardship, and policies for a healthier future
Source: Front Public Health. 2026 Jan 16;13:1726000. doi: 10.3389/fpubh.2025.1726000 (PMC12857316; doi:10.3389/fpubh.2025.1726000)
Supplement: Supplementary file 1 [file Table_1.docx]

**Fighting Antimicrobial Resistance in Brazil: Strengthening Diagnostic Stewardship, Antimicrobial Stewardship, and Policies for a Healthier Future**

**Marcelo Carneiro ^1, 2 *^, Marcelo Pillonetto ^3, 4 *^**

^1^School of Medicine, Department of Life Sciences; Postgraduate Program in Health Promotion, University of Santa Cruz do Sul; Medical leader on the Hospital Infection Control Committee and Coordinator of the Antimicrobial Stewardship Program at Santa Cruz Hospital, Brazil

^2^Scientific Coordinator of the Brazilian Association of Professionals in Infection Control and Hospital Epidemiology, Brazil

^3^School of Medicine and Health Sciences – Pontifical Catholic University of Paraná, Brazil

^4^Bacteriology Division, Central Laboratory of the State of Paraná, Brazil

*Both authors contributed equally to the paper as first and senior authors

**Supplemental Material**

**Brazil’s Strategic Response to Antimicrobial Resistance Through a One Health Approach**

Antimicrobial resistance (AMR) poses a significant and growing threat to global public health. In response, Brazil has developed a comprehensive national strategy, grounded in the One Health framework, which recognizes the interconnectedness of human, animal, and environmental health. The Brazilian National Action Plan for the Prevention and Control of Antimicrobial Resistance (PAN-BR AMR), aligned with the WHO Global Action Plan, seeks to strengthen surveillance systems, promote the rational use of antimicrobials, and enhance infection prevention and control practices across all relevant sectors. This multisectoral and integrated approach reflects the country's commitment to mitigating AMR through coordinated policies, evidence-based interventions, and sustained capacity-building within the Unified Health System (SUS). During the opening session of a recent national AMR forum, Ethel Maciel, Secretary of Health and Environmental Surveillance at the Ministry of Health, delivered the keynote address titled Antimicrobial Resistance: How to Change the Game by 2050? She presented current data on AMR-related mortality in Brazil and globally, and highlighted Brazil’s growing leadership in shaping global AMR governance. Secretary Maciel emphasized the role of the One Health paradigm in fostering cross-sectoral collaboration—from local to international levels—to tackle complex and emerging challenges such as pandemics, climate change, and AMR. She also detailed Brazil’s strategic priorities for the 2025–2030 cycle of PAN-BR AMR, which include implementing the plan in accordance with global political commitments; conducting research to estimate the prevalence of resistant pathogens in human, animal, and environmental reservoirs; promoting national, regional, and international cooperation and knowledge exchange; reducing antimicrobial use and its environmental impact; and reinforcing integrated surveillance and prevention strategies. These actions are designed to support Brazil’s contribution to global AMR governance through a cohesive and collaborative framework.

**Antimicrobial resistance surveillance: current outlook and future perspectives?**

Surveillance of antimicrobial resistance (AMR) is a critical component in the global effort to curb the spread of resistant pathogens and preserve the efficacy of antimicrobial therapies. Currently, they have been strengthening national surveillance networks that integrate data across human health, veterinary, and environmental sectors, in alignment with the One Health approach. Surveillance of AMR is a critical component of the global effort to curb the spread of resistant pathogens and preserve the efficacy of antimicrobial therapies. **Professor Dr. Carlos Kiffer**, from UNIFESP - Brazil and the vigiRAM project, expressed his enthusiasm in discussing the perspective of Brazilian national public health authorities. He emphasized the existing interface between applied research activities and AMR data in large territories such as Brazil, and even at the global level. “We live in an era of abundant data but limited information,” he noted. The integration of big data between academic institutions and public health managers remains a challenge, with a risk of missing the optimal timing to leverage these resources. The French experience, he argued, should serve as a model for Brazil and other countries. The use of national surveys to validate AMR epidemiological surveillance data can make the system both intelligent and pragmatic. Brazil is ready to undertake such studies. It is essential to optimize data generation—such as bacterial cultures and susceptibility profiles—produced by the thousands of laboratories across the country, both in human and veterinary health, which could serve as a national foundation for strategic interventions. Currently, Brazil is working to strengthen its national surveillance networks, which integrate data from the human health, animal health, and environmental sectors, in alignment with the One Health approach.

**Antimicrobial Resistance and Diagnostic Stewardship: Current Challenges and Future Perspectives**

Antimicrobial resistance (AMR) poses significant challenges to timely and accurate diagnosis, which is essential for guiding appropriate treatment and stewardship strategies. Conventional microbiological methods, while widely used, are often time-consuming and may delay targeted therapy, contributing to the empirical use of broad-spectrum antibiotics and the selection of resistant strains. In low- and middle-income countries, including Brazil, limitations in diagnostic infrastructure, laboratory capacity, and access to rapid testing technologies further complicate the effective management of infections. Emerging diagnostic tools—such as molecular assays, next-generation sequencing, and point-of-care testing—offer promising perspectives for early detection of resistant pathogens and resistance genes. However, their implementation requires investment, trained personnel, and system integration. Bridging these gaps is crucial for strengthening antimicrobial stewardship programs and improving clinical outcomes in the face of escalating AMR.

**Rational Antimicrobial Use: Impacts of the COVID-19 Pandemic and Best Practices for Combating Antimicrobial Resistance**

The COVID-19 pandemic has significantly affected global healthcare systems, exacerbating preexisting challenges related to AMR. During the early phases of the pandemic, widespread empirical use of antibiotics (broad-spectrum antibiotics) — often without confirmed bacterial co-infection—contributed to increased antimicrobial consumption and selective pressure, particularly in hospital settings. Simultaneously, disruptions in infection prevention programs, laboratory surveillance, and antimicrobial stewardship activities compromised the ability to monitor and respond to resistance patterns effectively. These developments have underscored the urgent need to strengthen AMR preparedness through the adoption of best practices, including enhanced diagnostic capacity, rational antimicrobial prescribing, robust surveillance systems, and intersectoral coordination, and during her lecture on addressing rising AMR rates in the context of COVID-19. **Dr. Twisha S. Patel**, Division of Healthcare Quality Promotion, National CDC, USA, emphasized that this global health crisis created opportunities to implement and strengthen best practices in ASPs, including preparations for future pandemics. She also highlighted the CDC’s G-ASET tool as a valuable resource for hospitals lacking a fully structured ASP, offering a feasible alternative to support stewardship initiatives in low-resource settings. Another critical point raised was derived from her research, which supports the notion that expanding access to antibiotics should be a global health priority, while simultaneously stressing the importance of “access without excess.” This concept, as emphasized by Patel, implies that antibiotic access must be intrinsically linked to high-quality diagnostics, professional clinical expertise, and well-functioning ASPs. Integrating these strategies into pandemic recovery efforts is essential to prevent further acceleration of AMR and to ensure resilience in future public health emergencies.

| **Conference: "Antimicrobial resistance: how to change the game by 2050?"** | | |
| --- | --- | --- |
|  |  |  |
| **Time** | **Topic** | **Speaker** |
| 14:00 - 14:10 |  | Emmanuel Lenain  (French Ambassador for Brazil) |
| 14:10 - 14:15 | Welcome | Alain Mérieux  (Mérieux Foundation) |
| 14:15 - 14:20 |  | Arnaud Favry  (P&GA BMX) |
| 14:20 - 14:40 | Main lecture: "The plan to combat antimicrobial resistance in Brazil" | Ethel Maciel (Secretary of Health and Environmental Surveillance - SVSA/MoH) |
| 14:40 - 15:00 | Lecture: "Resistance surveillance: the French Government's Viewpoint" | Pierre-Yves Bello (Global Health Advisor, French Government) |
| 15:00- 15:30 | Round table: Resistance surveillance: Where are we and where do we want to go? | Moderator: Marcelo Pilonetto  (CGLAB/LACENPR) Pierre-Yves Bello (French Government) Renata Peral (CGLAB/SVS/MoH)  Carlos Kiffer (UNIFESP) |
| 15:30 - 15:50 | Lecture: "Antimicrobial resistance: challenges and perspectives on diagnostic" | Ramanan Laxminarayan  (One Health Trust) |
| 15:50 - 16:20 | Round table: Diagnostic Steward- ship: where are we and where do we want to go? | Moderator: Renata Tigulini (CGLAB/SVS)  Samantha Serrano (One Health Trust) Viviane Dias (HNSG/ABIH)  Ana Paula Carvalho-Assef (Labsur) |
| 16:20 - 16:40 | Lecture: "Rational Use of Antimicrobials: The Impact of COVID-19 and Best Practices for Combating Antimicrobial Resistance" | Twisha Patel (CDC) |
| 16:40 - 17:10 | Round table: "Antimicrobial Stewardship: Where are we and where do we want to go? | Moderator: Marcelo Carneiro (ABIH) Twisha Patel (CDC)  Fábio Motta (HIPP)  Magda Costa (GVIMS/GGTES/ANVISA) |
| 17:10 - 17:30 | Q&A and closing | Marcelo Carneiro (ABIH) and Marcelo Pilonetto (CGLAB/LACENPR) |
